# Enhanced Defluoridation of Water Using Zirconium—Coated Pumice in Fixed-Bed Adsorption Columns

**DOI:** 10.3390/ma14206145

**Published:** 2021-10-16

**Authors:** Wondwosen Sime Geleta, Esayas Alemayehu, Bernd Lennartz

**Affiliations:** 1 School of Chemical Engineering, Jimma Institute of Technology, Jimma University, Jimma P.O. Box 378, Ethiopia; wondeto@gmail.com; 2 Faculty of Agricultural and Environmental Sciences, University of Rostock, Justus-Von-Liebig-Weg 6, 18059 Rostock, Germany; 3 Faculty of Civil and Environmental Engineering, Jimma Institute of Technology, Jimma University, Jimma P.O. Box 378, Ethiopia; 4 Africa Center of Excellence for Water Management, Addis Ababa University, Addis Ababa 1176, Ethiopia

**Keywords:** adsorption, defluoridation, fluoride, VPum, zirconium–coated pumice

## Abstract

Millions of people across the globe suffer from health issues related to high fluoride levels in drinking water. The purpose of this study was to test modified pumice as an adsorbent for the purification of fluoride-containing waters. The adsorption of fluoride onto zirconium-coated pumice (Zr–Pu) adsorbent was examined in fixed-bed adsorption columns. The coating of zirconium on the surface of VPum was revealed by X-ray diffractometer (XRD), Inductively coupled plasma-optical emission spectroscopy (ICP-EOS), and X-ray fluorescence (XRF) techniques. The degree of surface modification with the enhanced porosity of Zr–Pu was evident from the recorded scanning electron microscope (SEM) micrographs. The Brunauer-Emmett-Teller (BET) analysis confirmed the enhancement of the specific surface area of VPum after modification. The Fourier transform infrared (FTIR) examinations of VPum and Zr–Pu before and after adsorption did not reveal any significant spectrum changes. The pH drift method showed that VPum and Zr–Pu have positive charges at pH_PZC_ lower than 7.3 and 6.5, respectively. Zr–Pu yielded a higher adsorption capacity of 225 mg/kg (2.05 times the adsorption capacity of VPum: 110 mg/kg), at pH = 2 and volumetric flow rate (Q_O_) of 1.25 mL/min. Breakthrough time increases with decreasing pH and flow rate. The experimental adsorption data was well-matched by the Thomas and Adams-Bohart models with correlation coefficients (R^2^) of ≥ 0.980 (Zr–Pu) and ≥ 0.897 (VPum), confirming that both models are suitable tools to design fixed-bed column systems using volcanic rock materials. Overall, coating pumice with zirconium improved the defluoridation capacity of pumice; hence, a Zr–Pu-packed fixed-bed can be applied for defluoridation of excess fluoride from groundwater. However, additional investigations on, for instance, the influences of competing ions are advisable to draw explicit conclusions.

## 1. Introduction

Fluoride is among the many vital trace elements required in drinking water within the allowable range (<1.5 mg/L) [1] for the normal growth of humans and animal bones. Nevertheless, it is detrimental to bone development when ingested beyond the acceptable concentration limit (>1.5 mg/L) [2]. Excess fluoride hurts bones because of its high electronegative value, enabling interrelations with calcium in bones. Hence, it causes dental fluorosis and/or skeletal fluorosis (bone cancer) [3]. Alzheimer’s syndrome, arthritis, thyroid, etc., are additional adverse consequences of excess fluoride in drinking water [4]. 

In several places around the globe, groundwater is the principal and favored source of potable water, as is the case for many communities in rural and urban areas in the African rift valley. However, over 200 million people around the globe, including East Africa, ingest groundwater with high fluoride concentrations, which has a serious effect on people’s welfare [5,6]. Fluorosis is the most prevalent geochemical disease in the East African Rift, affecting over 80 million people [5,7,8]. Ethiopia is one of the East African countries with a large community residing where excess fluoride is becoming a significant concern, especially along the country’s central Rift [9,10]. The dissolution of fluoride-containing minerals has intensified excessive fluoride in the Ethiopian Rift in parent rocks and soils, which is usually linked to high bicarbonate and low calcium levels [11,12]. Levels of fluoride in wells in Ethiopia’s Rift are typically up to ten times higher than that of the WHO norm, putting millions of Ethiopians at risk of severe fluoride ion toxicity [9,13]. Similar to Ethiopia, countries like India, China, Pakistan, and others, deal with similar issues. Hence, excess fluoride in drinking water is among the most pressing issues the world faces today; therefore, valuable and easy-to-apply techniques to maintain fluoride concentrations within the allowable standard are urgently required.

Despite several fluoride removal methods, economic, procedural, and environmental disadvantages restrict their wide usage in many parts of the world. Although reverse osmosis [14,15], ultrafiltration [16], electrodialysis [17], and ion exchange [18,19,20] have good fluoride removal efficiency and are fairly steady, their operating costs are prohibitively high for developing countries like Ethiopia. The coagulation sedimentation methods [21,22,23,24] are simple to use, low cost and simple to apply; nevertheless, the dosage is too high and cannot be regenerated, causing secondary pollution and difficulty reducing fluoride to permissible levels. Adsorption using low-cost and locally available materials has been considered a highly efficient and well-accepted fluoride removal process by researchers of recent decades [25,26,27,28].

Adsorbent materials investigated for fluoride removal are numerous [26,29,30,31,32,33,34,35]. However, many of the materials suffer from either time-consuming synthesis procedures, high manufacturing costs, scarcity of raw materials, or a short lifetime, making them impractical to apply in remote regions of Africa. Subsequently, efforts have been made to acquire readily available, long-lasting, inexpensive, and effective materials that can be utilized to purify polluted water in low-income countries. Surface modification of locally available materials to treat fluoride-laden water is also under review since this could have a capacity for cost minimization and increasing sustainability. 

Pumice (VPum) is among the most encouraging and low-priced naturally available materials that have been broadly examined and applied for pollutant removal in water treatment [5,36,37,38]. Owing to the release of gasses during solidification, VPum has a light color and porous configuration. Good removal capabilities, mechanical strength, and absence of toxicity are the major benefits of VPum over other natural or synthetic adsorbents [37]. Many nations, including Spain, Greece, Turkey, Ethiopia, and Eritrea, have plenty of pumice deposits [5,36]. From the previous report, it is generally recognized that natural adsorbents modified with multivalent metal cations, such as Fe^3+^, Mn^4+^, and Zr^4+^, may change the surface properties and the affinity of fluoride [39]. Among these, zirconium (Zr^4+^) is paid more attention due to its non-toxicity, high binding affinity with fluoride ions, and acceptable cost [40,41]. Studies have found that surface-modified pumice might perform better as an adsorbent for water contaminants [42,43,44]. Hence, research into zirconium-based adsorbents with good performance should be considered.

With the above issues in mind, fixed-bed columns packed with zirconium (IV) oxychloride octahydrate (ZrOCl_2_.8H_2_O) coated pumice (abbreviated as Zr–Pu hereafter), which was not previously tested to treat fluoride contaminated water, the objectives of the present study were to (1) compare the defluoridation capacity of Zr–Pu and VPum in a fixed-bed column mode; (2) investigate the effect of solution pH and flow rate on the fluoride adsorption process; and (3) describe and analyze the adsorption processes employing well-known fixed-bed adsorption kinetic models such as the Thomas and Adams-Bohart model.

## 2. Materials and Methods

### 2.1. Adsorbent Preparations

#### 2.1.1. Natural Pumice

**Figure 1:**
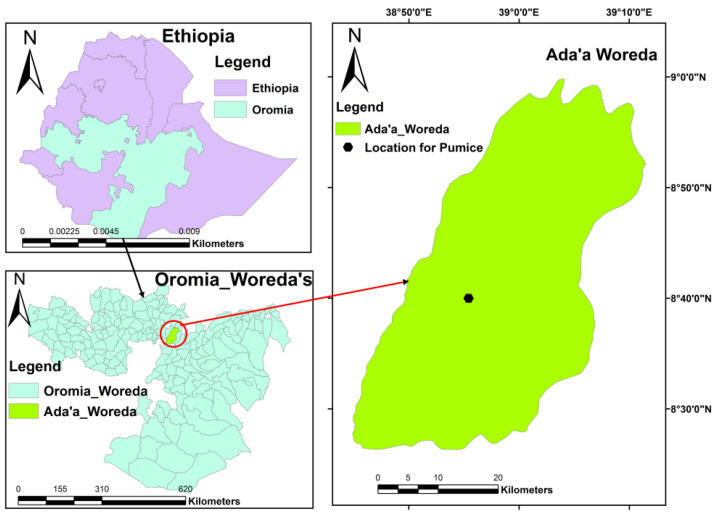
The geographical location of natural pumice (VPum) sample collections.

The natural pumice (VPum) used in this work was gathered from volcanic cones of the Main Rift Valley area of Oromia Regional State, East Showa Zone, Ethiopia, around 50–100 km East of Addis Ababa (Figure 1). VPum was repeatedly washed to eliminate possible attachments and other water-soluble substances from its surface. The washed VPum was dried in an oven at a temperature of 70 °C for 48 h, and subsequently, it was separated into four different mesh size fractions, as indicated in [5,45]. 

In our very recent study [5], VPum with a fine particle size (0.075–0.425 mm) revealed a good fluoride uptake performance compared to the other particle sizes. Hence, this fine particle size was used for coating with zirconium.

#### 2.1.2. Coating of VPum with Zirconium

The coating of VPum with zirconium was performed using 0.1 M ZrOCl_2_.8H_2_O in accordance with the methods applied by Salifu et al. [46], in which pumice was coated with aluminum (hydr) oxide. An adequate amount of ZrOCl_2_·8H_2_O solution was added to completely soak the dried pumice in acid-washed cylindrical polyethylene wide-mouth plastic bottles. The mixture was shaken with a horizontal shaker (SM25, Edmund Bühler 7400 Tübinger, Germany) at 200 rpm for 12 h. The zirconium-coated pumice (Zr–Pu) was decanted, dried in an electric oven at 70 °C for 48 h, and soaked in 2 M NH_4_OH. The Zr–Pu was washed repeatedly with deionized water, dried at 70 °C for 48 h, and stored in a plastic bag for subsequent experiment and characterization.

### 2.2. Chemicals and Reagents

All bottles and glassware were properly cleaned and rinsed with deionized water before use. All chemicals and reagents used in the experiments were of analytical quality. Zirconium oxychloride (IV) octahydrate (ZrOCl_2_·8H_2_O), ammonium hydroxide (NH_4_OH), sodium hydroxide (NaOH), and hydrochloric acid (HCl) were purchased from Merck KGaA, Darmstadt, Germany. A 1000 mg/L fluoride stock solution was made by dissolving 2.21 g of NaF in 1000 mL of deionized water and stored at 4 °C in a refrigerator. The synthetic solution for fixed-bed adsorption experiments was prepared by diluting the stock solution with deionized water to obtain the desired concentration. NaOH (0.1 M) and/or HCl (0.1 M) solutions were added to adjust the pH values of the fluoride solution. 

### 2.3. Adsorbent Characterizations

#### 2.3.1. Crystalline Structure

The crystalline structure of VPum and Zr–Pu before and after the adsorption experiment was determined by an X-ray diffractometer (XRD-7000, Drawell, Shanghai, China) with Cu Kα as a radiation source (1.54056 Å) generated at 30 kV using a 25 mA instrument. The diffractogram was obtained with a step width of 2θ and a scan rate of 0.01°/min. The mineralogical buildup of the adsorbents was illustrated by contrasting the X-ray diffractogram with the database of the X’pert HighScore Plus software package (Version: 2.2b 2.2.2). 

#### 2.3.2. Chemical Composition

The inductively coupled plasma-optical emission spectroscopy (ICP-OES) was employed to examine the elemental composition of the VPum and Zr–Pu. The oxide contents of VPum and Zr–Pu were analyzed by X-ray fluorescence (XRF) spectroscopy.

#### 2.3.3. Fourier Transform Infrared (FT-IR) Analysis

The Fourier Transform Infrared (FT-IR) analysis was run on KBr pellets to learn about the different functional groups of the samples. The spectra were recorded over a range of 5000 to 400 cm^−1^ at a resolution of 0.1 cm^−1^ in a PerkinElmer spectrometer (UNSW Sydney, Australia) using a lithium tantalite (LiTaO_3_) detector.

#### 2.3.4. Surface Area (S_BET_) and Pore-Size Distribution Analysis 

The Brunauer-Emmett-Teller (BET) was used to analyze the surface area (*S_BET_*) of the adsorbents. Barrett–Johner–Halenda (BJH) equation was used to determine the pore size distribution of the adsorbents. 

#### 2.3.5. Scanning Electron Microscope (SEM) Analysis

A scanning electron microscope (SEM) (JCM-6000plus, Version 0.2, JEOL Ltd., Peabody, MA, USA), operated at 15 kV, was used to determine the morphologies of VPum, Zr–Pu before and after adsorption. 

#### 2.3.6. pH and Point of Zero Charges (pH_PZC_)

The pH of VPum, Zr–Pu before and after adsorption was determined using a pH meter in a 1:10 adsorbent/water ratio as per the standard method [5,47]. The point of zero charges (pH_PZC_) of the adsorbents was determined by the pH drift method [5,48], using 0.01 M of NaCl solutions as an electrolyte and adding 0.1 M of NaOH or HCl solutions.

#### 2.3.7. Surface Acidity/Basicity Analysis

Boehm’s titration method [49] was used to determine the surface acidity/ basicity of VPum and Zr–Pu. A dried adsorbent sample (0.1 g) was mixed with 50 mL of 0.05 M NaOH or 0.05 M HCl under the N_2_ atmosphere. The samples were shaken for 2 h and then filtered to remove the adsorbent. The excess base and acid were titrated with 0.05 M HCl and 0.05 M NaOH, respectively. The acidity and basicity of the surface were determined assuming that HCl and NaOH neutralize all basic groups and acidic groups, respectively.

### 2.4. Fixed-Bed Column Adsorption Studies

The fluoride adsorption capacity of Zr–Pu for fluoride was evaluated by continuous up-flow column systems, as indicated in [5]. A small-scale cylindrical filter column with an inner diameter of 8.1 cm and a height of 10 cm was used to conduct continuous up-flow adsorption experiments. A weighted amount of Zr–Pu was packed uniformly with care into the column as a fixed-bed absorber. One pore volume of deionized water was passed through the bed to avoid the potential occurrence of voids, channeling, or cracking. The fluoride solution as influent was pumped into the fixed-bed column in up-flow mode by a variable flow peristaltic pump (REGLO ICC, Ismatec, Cole-Parmer Barrington, IL, USA). All experiments were performed at a temperature of 298 K. An automatic fraction collector (RFI, MA-RON GmbH, Reichelt Chemietechnik GmbH + Co., Heidelberg, Germany) was used to collect effluent samples at the outlet of the column set-up. Ion chromatography (930 Compact IC Flex, Metrohm, Herisau, Switzerland) was employed to measure the concentration of fluoride in the effluent samples. The desired breakthrough concentration (C_b_) was considered to be 1.5 mg/L [50]. The exhaustion point was characterized as the point at which the fluoride concentration in the effluent was equal to 90% of the fluoride concentration in the influent (i.e., 0.9 C_t_/C_O_).

The influence of experimental variables such as the pH of the feed solution (2, 4, and 6) and influent volumetric flow rate (1.25, 2.50, and 3.75 mL/min) on the shape of the breakthrough curves and amount of fluoride removed by Zr–Pu were tested at a constant fixed-bed column height of 10 cm and an initial fluoride concentration of 10 mg/L. The defluoridation performance of Zr–Pu was compared with the performance of VPum presented in our very recent study [5]. The plots of experimental breakthrough curves were displayed only for Zr–Pu. 

### 2.5. Analysis of Column Data

The shape of the breakthrough curve is an essential feature for describing the adsorption capacity of the adsorbent in a flow-through column. The breakthrough curves and breakthrough time (t_b_) are the characteristic features resulting from the adsorption dynamics and process design of the packed-bed column. These two parameters have a significant effect on the feasibility and economics of the adsorption process [5,51]. The breakthrough parameters are influenced by the experimental conditions of the experiment, such as the initial flow rate and pH of the influent solution. The breakthrough curve is stated as the ratio of effluent to influent fluoride concentrations (C_t_/C_O_) as a function of time. The exhaustion or saturation time and the breakthrough time are expressed by Equations (1) and (2), respectively.

(1) $$t_{e}=\int_{t=0}^{t=t_{\mathrm{total}}}\left(1-\frac{C_{t}}{C_{o}}\right)\mathrm{dt}$$

(2) $$t_{b\;}=\int_{t=0}^{t_{b}}\left(1-\frac{C_{t}}{C_{o}}\right)\mathrm{dt}$$

where, t_e_ is the saturation time (min), and t_b_ is the breakthrough time (min) at which C_t_ = C_b_ (mg/L) (for the current system, C_b_ = 1.5 mg/L).

The total amount of fluoride adsorbed (q_total_: mg) in the column for a given feed concentration (C_t_), and initial flow rate (Q) can be obtained from Equation (3) [52].

(3) $$q_{\mathrm{total}\;}=\frac{\mathrm{QA}}{1000}=\frac{Q\times C_{O}}{1000}\int_{t=0}^{t=t_{\mathrm{total}}}\left(1-\frac{C_{t}}{C_{O}}\right)\mathrm{dt}$$

where Q (mL/min) is the volumetric flow rate, and ‘A’ is the area under the breakthrough curve. 

The equilibrium fluoride uptake capacity (q_e:_ mg kg^−1^) of the packed-bed column is determined by dividing the total amount of fluoride adsorbed (q_total_) by the amount of dry mass of the adsorbent used (m), Equation (4).

(4) $$q_{\mathrm{eq}\;}=\frac{q_{\mathrm{total}}}{m}=\frac{C_{o}{\mathrm{Qt}}_{e}}{m}$$

The experimental uptake capacity or amount of fluoride removed at t_b_ (q_b_: mg kg^−1^) can be calculated from Equation (5).

(5) $$q_{b\;}=\frac{C_{o}{\mathrm{Qt}}_{b}}{m}$$

The volume of effluent (V_e_) and the volume of treated effluent or breakthrough volume (V_b_) of solution can be calculated from Equations (6) and (7), respectively.

(6) $$V_{e}={\mathrm{Qt}}_{e\;}$$

(7) $$V_{b}={\mathrm{Qt}}_{b}$$

where V_e_ is the total volume of the effluent until exhaustion/saturation time (mL) and V_b_ is the total volume of the effluent until the breakthrough time (mL). 

The Mass Transfer Zone (MTZ) or unused bed length (H_UNB_) can be obtained from Equation (8) [53].

(8) $$\mathrm{MTZ}=H_{T}\left(\frac{t_{e}-t_{b}}{t_{e}}\right)$$

where H_T_ is total bed height (cm), t_e_ (min) is exhaustion time, and t_b_ is breakthrough time (min).

The Empty Bed Contact Time (EBCT) is defined as the time of contact between the adsorbent and the adsorbate solution, which can be evaluated from Equation (9).

(9) $$\mathrm{EBCT}=\frac{V_{B\;}}{Q}$$

where V_B_ (mL) and Q (mL/min) designate the bed volume and the influent flow rate, respectively.

### 2.6. Breakthrough Curve Modeling

Before upscaling the study to a manufacturing application, the column data should be validated with theoretical modeling. Different kinetic models have been tested for estimating the breakthrough performance of fixed-bed column adsorption systems [54,55]. Besides, these kinetic models have been employed to evaluate the kinetic column parameters and uptake capacity of the column. In this work, to describe the dynamic behavior of fluoride adsorption using Zr–Pu in a fixed-bed column filter, the two most important and widely used mathematical models, the Thomas model and Adams-Bohart model, were applied to the experimental data. The acquired model parameters for the defluoridation of water were compared with the Thomas model and Adams-Bohart model parameters obtained for the defluoridation of water onto VPum from our recent work [5].

#### 2.6.1. Thomas Model

The Thomas model is among the most common and highly employed dynamic models in a fixed-bed column performance operation. The model assumes that the process obeys Langmuir kinetics of adsorption-desorption with no axial dispersion, and the driving force obeys second-order reversible reaction kinetics [56]. 

The Thomas model can be used to determine the maximum uptake of adsorbate and adsorption rate constants for the continuous adsorption process [56]. The non-linear form of the Thomas model is given by Equation (10).

(10) $$\frac{C_{t}}{C_{o}}=\frac{1}{1+\exp\left[K_{T}q_{o}\frac{m}{Q}-K_{T}C_{o}t\;\right]}$$

where K_T_ is the Thomas rate constant (L/min mg), C_o_ (mg/L) is the inlet or initial concentration, C_t_ (mg/L) is the effluent fluoride concentration at the time, t, Q (L/min) is the flow rate, q_o_ (mg/kg) is the equilibrium adsorbate uptake, and m (kg) is the amount of adsorbent (dry mass) in a fixed-bed.

#### 2.6.2. Adams-Bohart Model

The Adams–Bohart model [57] was developed to analyze the dynamics of fixed-bed systems. It is based on the hypothesis that the adsorption rate is related to both the residual adsorbent and adsorbate concentration. For the estimation of breakthrough curves and model parameters, the non-linear form of the Adams–Bohart model (Equation (11)) [58] was applied.

(11) $$\frac{C_{t}}{C_{o}}=\frac{1}{1+\exp\left[K_{\mathrm{AB}}N_{o}\frac{Z}{v}-K_{\mathrm{AB}}C_{o}t\;\right]}$$

where K_AB_ (L/mg. min) is the kinetic constant, *v* (mL/min) is the linear flow rate, Z (cm) is a column bed depth, N_O_ (mg/L) is the saturation concentration, and time t (min) ranges from the start to breakthrough points for fluoride. The linear flow rate (superficial velocity) was determined by Equation (12).

(12) $$v=\frac{Q}{A}$$

where A is the cross-sectional area of the fixed-bed (cm^2^), and Q is the volumetric flow rate (mL/min).

The root-mean-squared error (RMSE) (Equation (13)) was used to measure the differences between the results predicted by the models and experimental data.

(13) $$\mathrm{RMSE}=\sqrt{\frac{1}{N}\overset{N}{\underset{i=1}{\sum }}{\left(\mathrm{predicted}\;\mathrm{value}-\mathrm{experimental}\;\mathrm{value}\right)}^{2}}$$

where N is the number of data points.

## 3. Results and Discussions

### 3.1. Characterization of Adsorbents

#### 3.1.1. Crystalline Structure

**Figure 2:**
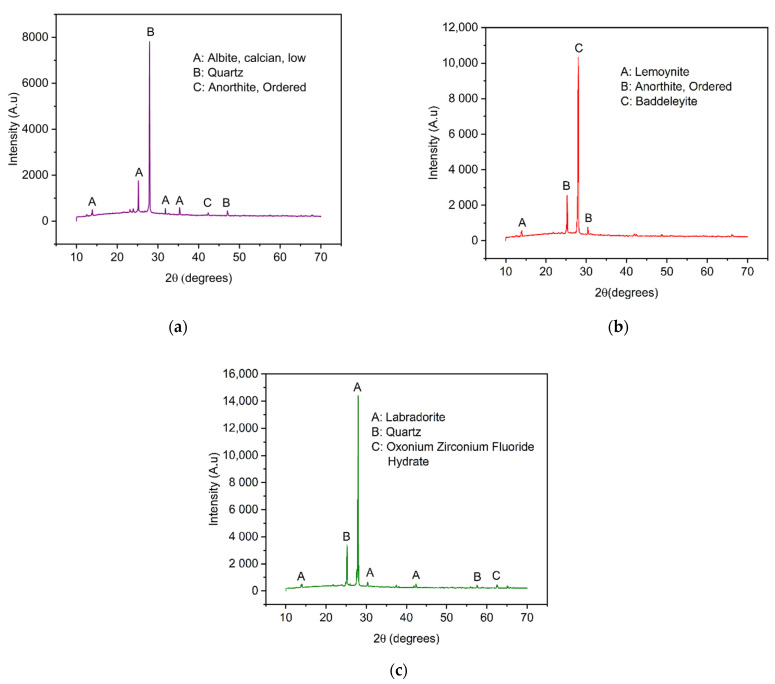
XRD patterns for (**a**) VPum and Zirconium–coated pumice (Zr–Pu) (**b**) before and (**c**) after adsorption.

The crystalline structure of VPum and Zr–Pu before and after adsorption was explored by X-ray diffraction (XRD), as presented in Figure 2. The results showed that the dominant crystalline phases of VPum are silicon oxide (SiO_2_) and Anorthite, Ordered (CaAl_2_Si_2_O_8_). Lemoynite (Na_2_CaZr_2_Si_10_O_26_(H_2_O)), Baddeleyite (ZrO_2_), and Anorthite, Ordered (CaAl_2_Si_2_O_8_) are the main crystalline components of Zr–Pu before adsorption, while quartz (SiO_2_), Labradorite (Na_.5_Ca_.5_Al_1.5_Si_2.5_O_8_), and Oxonium Zirconium Fluoride Hydrate (ZrF_5_H_3_O(H_2_O)_2_) were the dominant components of Zr–Pu after adsorption. The existence of crystalline phases in VPum (Figure 2a) can be deduced from the peaks at 2θ = 13.88°, 25.32°, 28.12°, 31.83°, 35.30°, 42.34°, and 42.34°. The XRD patterns of Zr–Pu before adsorption (Figure 2b) showed peaks at 2θ = 13.99°, 25.39°, 27.99°, and 30.38°; while the peaks at 2θ = 13.96°, 25.22°, 27.94°, 30.19°, 42.36°, 57.53°, and 62.48° were observed for Zr–Pu after adsorption (Figure 2c). VPum (Figure 2a) had a main peak at 28.12° (2θ), which had the highest intensity corresponding to SiO_2_ (peak B). Zr–Pu before adsorption (Figure 2b) had a main peak at 2θ = 27.99°, corresponding to ZrO_2_ (peak C) formed at the surface; while Zr–Pu after adsorption (Figure 2c) had a major peak at 27.95° (2θ), corresponding to Na_.5_Ca_.5_Al_1.5_Si_2.5_O_8_ (peak C). It is also worth noting that, in Figure 2b, the peaks of lower intensity observed in VPum at 35.30°, 42.34°, and 42.34° were almost undetectable in the XRD technique after the modification. This could be due to the growth of zirconium oxide over the surface [26]. Furthermore, some small peaks were detected after fluoride adsorption (Figure 2c). This could be because of a slight change in the structural framework of the adsorbent. 

#### 3.1.2. Chemical Composition

The chemical analysis revealed that the major elements in VPum were Si, Al, K, and Fe (Table 1). Other elements were available in limited fractions or were below the instrument’s detection limit. 

Through XRF measurement, the main components of VPum are oxides of Si, Fe, K, and Al. A previous study has reported comparable values for natural pumice [59].

The average amount of zirconium coated onto VPum was 3.9% (wt). The XRF measurement designated that 8.9% (wt) (Table 1) zirconium oxide was coated on VPum, enabling it to enhance its fluoride removal capacity.

#### 3.1.3. Fourier Transform Infrared (FTIR) Analysis

**Figure 3:**
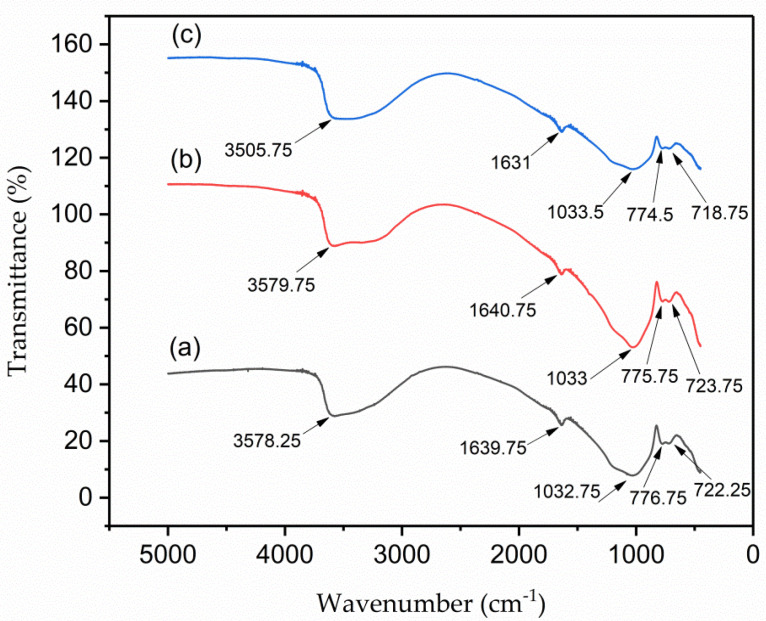
Fourier−transform infrared (FT−IR) for (**a**) VPum; Zr–Pu (**b**) before and (**c**) after adsorption.

The FTIR spectrums of VPum, Zr–Pu before adsorption and after adsorption at wavelengths between 400 cm^−1^ and 5000 cm^−1^ are shown in Figure 3a–c, respectively. Because of the Si–O–Si symmetric stretching vibration, the absorption band at ~1033 cm^−1^ can be attributed to the (SiO_4_)^2−^ groups. The peaks at ~775.75 and ~723.75 cm^−1^ belong to the stretching and bending vibrations of the Si–O groups. Some peaks, like the broadening peak at ~3579.75 cm^−1^ that belongs to the asymmetric stretching vibration of the H–O bond, can be allocated to adsorbed water molecules, and the peak at ~1640.75 cm^−1^ can be allotted to the bending vibration of the H-–O–H bond. In general, the IR spectrum of the natural pumice, Zr–Pu, before and after adsorption appeared approximately similar and consistent with those reported in previous studies [60,61].

#### 3.1.4. Surface Area (S_BET_) and Pore-Size Distribution Analysis

The BET specific surface area (S_BET_) of VPum and Zr–Pu was 3.45 and 9.63 (m^2^/g), respectively. It was observed that coating with zirconium enhanced the specific surface area of VPum (Table 2). A similar observation was made in the removal of pollutants using cerium-loaded pumice [62]. Based on the BJH method, the calculated pore size distribution resulted in an average pore size of 4.43 nm for VPum and 3.52 nm for Zr–Pu. This result showed that the adsorbents are a mesopore material by the IUPAC classification. As can be observed from Table 2, the pore size of VPum was changed after modification. The change in pore size reveals that zirconium oxide reached the internal pore. The ionic radius of fluoride is 0.133 nm, which is much smaller than the average pore size of VPum and Zr–Pu, confirming that the fluoride ions can easily penetrate the inner layers of the adsorbents.

#### 3.1.5. Scanning Electron Microscope (SEM) Analysis

**Figure 4:**
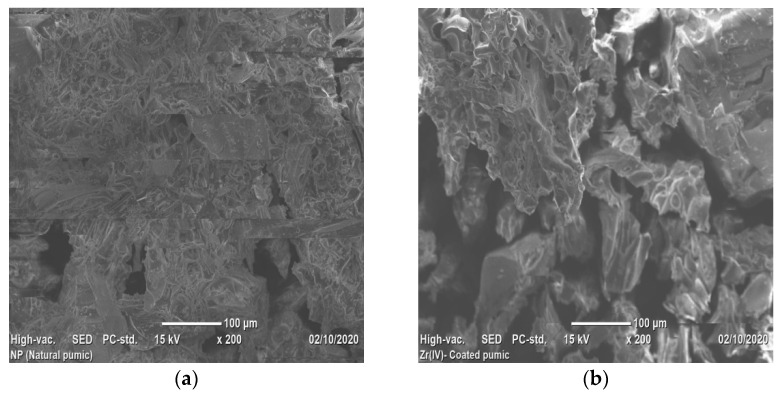

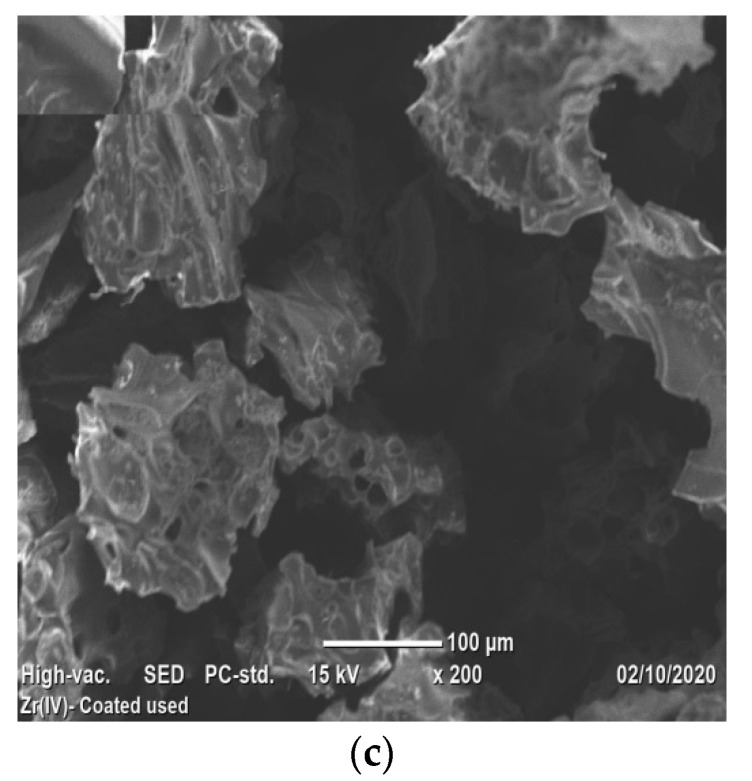
SEM micrographs of (**a**) VPum; Zr– Pu (**b**) before and after (**c**) adsorption.

Scanning Electron Microscope analysis displayed the morphology of VPum and Zr–Pu before and after adsorption (Figure 4a–c). Figure 4a shows that VPum had an irregular texture with a rough surface and some pores. As seen from the SEM images of Zr–Pu before (Figure 4b) and after (Figure 4c) adsorption, the surface of the VPum was changed. The surface of Zr–Pu became dense, and channels/pores can be noticed. The dense surface was attributed to the exterior surface of VPum, which was coated with zirconium. At the same time, the formation of pores originated from further removal of water-soluble compounds and dust by washing it repeatedly with deionized water. The improvements of the porous structure and adsorption for surface modification of pumice and salt treatment of zeolite were reported by Sepehr et al. [43] and Liang and Ni [63], respectively. As seen from Zr–Pu’s micrographs after adsorption (Figure 4c), the pore morphology was altered, which produced a large but limited number of heterogeneous channels. This could elucidate the slight reduction in particle structure of VPum during modification and adsorption processes. Similar remarks were also drawn in a previous study [63].

#### 3.1.6. pH and Point of Zero Charges (pH_PZC_)

The pH in water and pH_PZC_ of VPum were found to be 8.8 and 7.3, respectively. These pH values are very close to values reported previously for VPum [5]. The pH in water and pH_PZC_ for Zr–Pu were identified as 7.7 and 6.5, respectively. In the current study, both the pH in water and the pH_PZC_ of Zr–Pu were found to be lower than that of VPum. A similar observation was reported for chitosan-pumice blends [64]. The adsorbent’s surface charge was positive when the pH of the solution was below pH_PZC_ ((Zr)–Pu (6.5), VPum (7.3)). When the pH is lower than pH_PZC_, fluoride can be adsorbed onto the surface of the adsorbents due to coulombic attraction [5,65]_._

#### 3.1.7. Surface Acidity/Basicity Analysis

The acid-base character of VPum and Zr–Pu was obtained from Boehm’s titration method. Acidity values of 0.805 and 0.875 (mmol/g) and basicity of 0.305 and 0.325 (mmol/g) were obtained for VPum and Zr–Pu, respectively. The notable observation is that VPum exhibit relatively lower acidity and basicity characteristics than Zr–Pu.

### 3.2. Effect of Experimental Conditions on Fluoride Removal

#### 3.2.1. Initial Solution pH

**Figure 5:**
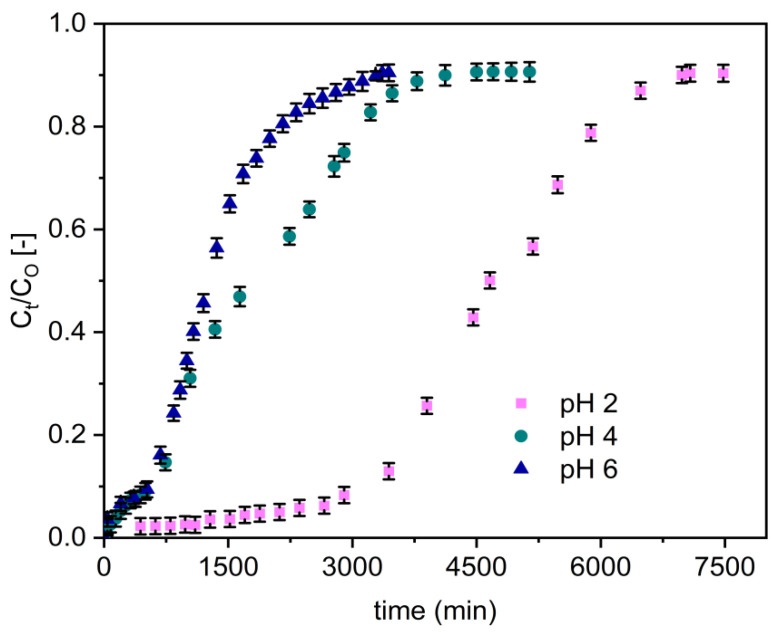
Effect of solution pH on the breakthrough performance of fluoride onto Zr–Pu (initial fluoride concentration 10 mg/L (C_O_: 10 mg/L); initial flow rate 1.25 mL/min (Q_O_: 1.25 mL/min); bed depth 10 cm).

The variation in defluoridation capacity of the adsorbents with respect to pH was evaluated at various pH values (2, 4, and 6) by a separate set of fixed-bed adsorption columns. The fluoride breakthrough curves obtained for Zr–Pu are presented in Figure 5 for a fixed inlet flow rate of 1.25 mL/min, influent fluoride concentration of 10 mg/L, and column bed depth of 10 cm. Sharper and earlier breakthrough curves emerged as the pH was raised from 2 to 6 (Figure 5).

The column adsorption parameters obtained for uptake of fluoride onto Zr–Pu, and VPum are presented in Table 3. Reduced mass transfer zone values were found at a low pH level (pH 2), resulting in longer breakthrough and exhaustion times. Furthermore, a lower solution pH (pH 2) improved column performances, increased treated water volume, improved defluoridation efficacy, and enhanced adsorption capability at breakthrough and exhaustion time. The highest fluoride uptake capacity was 225 mg/kg by Zr–Pu, whereas the fluoride uptake was 110 mg/kg by VPum. This showed Zr–Pu removed 2.05 times fluoride compared to the VPum. The breakthrough capacity of Zr–Pu was 163 mg/kg (3.02 times that of VPum (54 mg/kg)). A breakthrough time of 3471 min for Zr–Pu and 1206 min for VPum; and an exhaustion time of 4781 min for Zr– Pu and 2339 min for VPum were achieved at a pH of 2. Hence, VPum has the quickest time to breakthrough and exhaustion, while Zr–Pu had the longest time to breakthrough and exhaustion and a better adsorption capacity. When the initial pH values of the solution were greater than two, the fluoride removal rate for Zr–Pu, and VPum decreased (Table 3). At pH values of four and six, the decrease in the fluoride uptake may be ascribed to the decrease in the amount of H^+^ or HF adsorption because of electrostatic attraction [5,66]. The higher uptake capacity at pH 2 could be attributed to the fact that the adsorbent surface has more positive charges at lower pH and electrostatically adsorbs fluoride ions [66]. Hence, the adsorption of fluoride ions was due to an electrostatic phenomenon and surface complexation, which can occur alone or in combination with the fluoride ion’s uptake on the adsorbents. In general, Zr–Pu, and VPum showed similar pH-dependent fluoride uptake performances. However, a noticeable performance enhancement was seen due to the zirconium coating, primarily because of the specific interaction between fluoride ions and zirconium (hydr) oxide. A similar observation was made in the removal of pollutants using cerium-loaded volcanic rocks [62].

The breakthrough time was longer, and the volume of treated water was high at pH 2 for Zr–Pu. Accordingly, the pH of the solution was maintained at pH 2 in the subsequent experiment.

#### 3.2.2. Flow Rate

**Figure 6:**
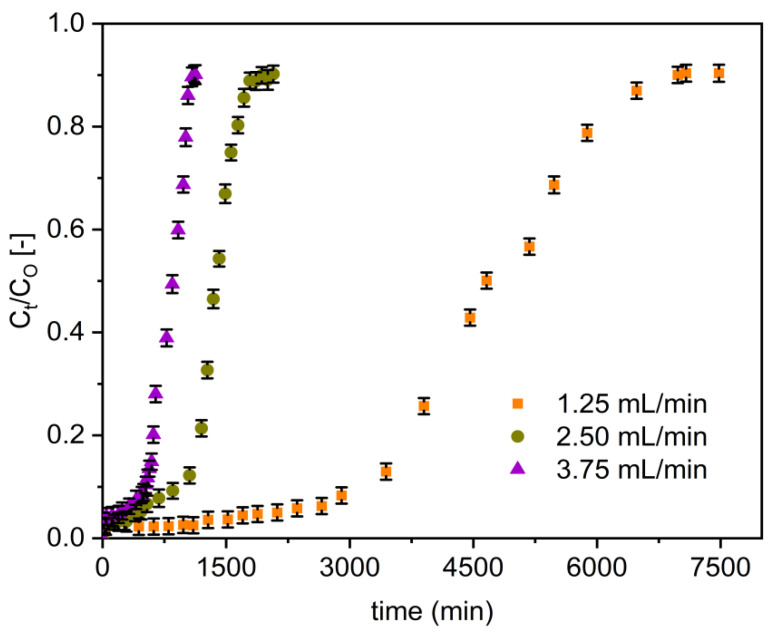
Effect of initial flow rate on the breakthrough performance of fluoride onto Zr–Pu (pH 2; C_O_: 10 mg/L; bed depth 10 cm).

Figure 6 presents the effect of various flow rates (1.25, 2.50, and 3.75 mL/min) on the fluoride breakthrough curves of Zr–Pu. When the flow rate increased from 1.25 to 3.75 mL/min, the breakthrough curves became steeper and occurred earlier (Figure 6). The breakthrough data displayed in Table 3 also confirmed that the flow rate rose from 1.25 to 3.75 mL/min resulted in the reduction of breakthrough time from 3471 to 557 min for Zr–Pu and from 1206 to 75 min for VPum. The exhaustion time also reduced from 4781 to 768 min and from 2339 to 359 min, Zr–Pu and VPum, respectively. At higher flow rates (higher hydraulic loading), the solution residence period in the column was shorter, hence less contact time between the adsorbate and adsorbent. The adsorbate ions exited the column before the equilibrium adsorption was reached, resulting in limited fluoride ion uptake. This assertion was backed up by MTZ (Table 3), which increases with increasing flow rate but narrows the utilized fractional bed [67]. Thus, the column’s maximum defluoridation capacity (q_e_) decreased from 225 to 108 mg/kg for Zr–Pu and 110 to 17 mg/kg for VPum with the rise in flow rate from 1.25 to 3.75 mL/min. Similarly, the defluoridation capacity at breakthrough time (q_b_) was reduced from 163 to 79 mg/kg for Zr–Pu and 54 to 7 mg/kg for VPum as the flow rate increased from 1.25 to 3.75 mL/min. The reduction in Empty Bed Contact Time (EBCT) values can also be observed in Table 3. Similar observations were made in other studies [6,68]. High flow rates reduced the contact time between the fluoride ions and the adsorbent surface, less fluoride was adsorbed, and the overall adsorption performance decreased. Although the influence of initial flow rate was similar for both Zr–Pu, and VPum, the zirconium coating resulted in a generally enhanced uptake of fluoride. 

The best column performance was seen at the lowest flow rate (1.25 mL/min); consequently, all experiments except for the effect of flow rate were done at a flow rate of 1.25 mL/min.

Overall, the variation of column parameters, such as q_e_, q_b_, V_e_, and V_b_, obtained for fluoride removal onto Zr–Pu and VPum at experimental parameters showed that Zr–Pu has more activity than VPum towards fluoride (Table 3). The coating of natural pumice with zirconium could account for the improved activity and, hence, adsorption capacity. 

### 3.3. Application of the Thomas Model

**Figure 7:**
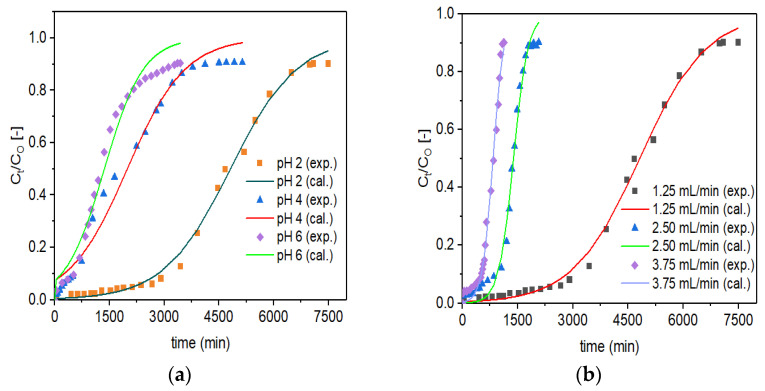
Experimental (exp.) and simulated (cal.; Thomas model) breakthrough curves of fluoride for Zr–Pu at different (**a**) pH values (C_O_: 10 mg/L; Q_O_: 1.25 mL/min; bed depth 10 cm) and (**b**) initial flow rates, Q_O_ (pH 2; C_O_: 10 mg/L; bed depth 10 cm).

The Thomas model (Equation (10)) was fitted non-linearly to the experimental data (designated as exp.) for Zr–Pu at various initial pH values (Figure 7a) and initial flow rates (Figure 7b). For VPum, the according figures are not shown in this study but were reported previously [5]. The Thomas model parameter values for the uptake of fluoride onto Zr–Pu, and VPum are summarized in Table 4. The slope and intercept data were used to evaluate the values of K_T_ and q_o_ for Zr–Pu and VPum. As the flow rates increased, the values of K_T_ increased, while the values of q_o_ decreased, implying that the Empty Bed Contact Time (EBCT) decreased. A decrease in q_o_ with increased flow rates resulted from a reduced interaction time among fluoride ions and adsorption sites [69,70]. As can be seen from Table 4, both adsorbents exhibited good adsorption capacity at pH 2, where K_T_ has the lowest value with 1.110 (L/min.mg) (×10^4^) for Zr–Pu and 1.44 (L/min.mg) (×10^4^) for VPum. The K_T_ value of Zr–Pu is smaller than that of VPum, revealing that the diffusion mass transfer is greater for Zr–Pu than VPum [71]. Nevertheless, the column adsorption capacity (q_o_) decreased from 226 to 64 (mg/kg) for Zr–Pu and from 110 to 13 (mg/kg) for VPum as the pH value increased from 2 to 6 while the other conditions were kept constant (Table 4). Overall, the Thomas model adequately represented the breakthrough data for defluoridation by both adsorbents at various experimental conditions as indicated by the R^2^ values (Zr–Pu: 0.980–0.995; and VPum: 0.953–0.995). The values obtained by the Thomas model optimization confirmed that the zirconium coating enhanced the fluoride uptake capacity.

### 3.4. Application of the Adams-Bohart Model

**Figure 8:**
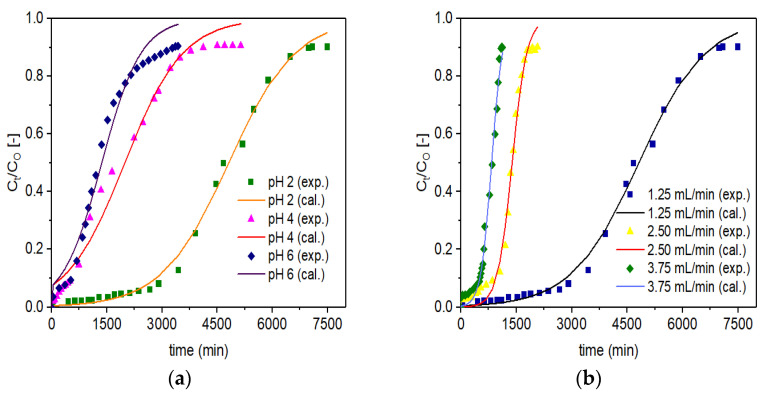
Experimental (exp.) and simulated (cal.; Adams-Bohart model) breakthrough curves of fluoride for Zr–Pu at different (**a**) pH values (C_O_: 10 mg/L; Q_O_: 1.25 mL/min; bed depth 10 cm) and (**b**) Q_O_ (pH 2; C_O_: 10 mg/L; bed depth 10 cm).

The plots for experimental (designated as exp.) and simulated (designated as cal.) breakthrough data based on the Adams-Bohart model are presented in Figure 8. Similar to the Thomas Model, the graphical plots for experimental data and simulated data are not shown for VPum, which were presented in our recent work [5]. The optimized model parameter values such as K_AB_ and N_O_ are summarized in Table 5. Like the Thomas model, it can also be possible to deduce that the increase in K_AB_ with increased flow rates or decreased EBCT decreases the values of N_O_. A decrease in N_O_ with increasing flow rates was attributed to EBCT reduction due to the direct proportion of the adsorption capacity to the interaction time. Similar observations were reported in previous studies [69,71]. Similar to the Thomas model, at pH 2, with a lower K_AB_ value (1.110 (L/min·mg) (×10^4^) for Zr–Pu and 2.568 (L/min·mg) (×10^4^) for VPum), both materials showed good adsorption properties (Table 5).

The lower value of K_AB_ for Zr–Pu than VPum suggests that the mass transfer by diffusion within the column packed with Zr–Pu was superior to VPum [71]. However, it is clear from Table 5 that the values of N_O_ decreased from 117 to 33 mg/L for Zr–Pu and from 46 to 8 mg/L for VPum as the pH value increased from 2 to 6, while the other conditions remained constant. 

The values of R^2^ range from 0.980 to 0.996 for Zr–Pu and 0.896 to 0.996 for VPum, indicating the general applicability of the Adams-Bohart model for describing the experimental data (Table 5). The Adams-Bohart model did a good job of depicting the adsorption process and defluoridation by Zr–Pu and VPum packed fixed–beds at various experimental conditions. Similar to the Thomas model, the optimized model parameter values confirmed that zirconium coating improved the fluoride uptake capacity of VPum.

In general, the Thomas and Adams-Bohart models described the experimental data very well, revealing that the models are suitable tools for designing fixed-bed column systems using VPum and Zr–Pu. 

### 3.5. Performance of various Adsorbents on Fluoride Uptake

The adsorbent (Zr–Pu) utilized in this work was compared to previously studied adsorbents for uptake of fluoride in a flow-through fixed-bed column system, as shown in Table 6.

The fluoride uptake capacity of Zr–Pu used in this study is higher than those of granular acid-treated bentonite, aluminum modified iron oxide, VPum, and VSco. Above all, the raw material (VPum) is easily accessible and readily available, in contrast to some of the other substrates, confirming that Zr–Pu could be a promising candidate for the uptake of excess fluoride from water.

## 4. Conclusions

In this study, the defluoridation performance of the Zr–Pu-packed fixed-bed column systems were examined and compared with that of VPum. The XRD analysis showed that zirconium oxide was coated on the surface of VPum. The enhancement of the specific surface area was confirmed by the BET technique. The degree of surface modification with the improved porosity of Zr–Pu was evident from the recorded SEM image. The ICP-OES and XRF analysis were conducted for VPum and Zr–Pu to confirm the absence of harmful substances and to quantify the amount of zirconium coated onto the VPum. Fluoride adsorption was influenced by pH and input flow rate. The highest uptake capacity of fluoride by Zr–Pu was 225 mg/kg (2.05 times that of VPum: 110 mg/kg), at pH 2, input fluoride concentration of 10 mg/L, and input flow rate of 1.25 mL/min. A fixed-bed column of 265 g Zr–Pu can generate a volume of 4339 mL (~2.9 times that of VPum: 1508 mL) treated water with an acceptable fluoride level of < 1.5 mg/L. Such enhanced performance is most likely associated with the coating of VPum with zirconium. The Thomas and Adams–Bohart models were employed to evaluate the breakthrough curves and obtain values for the kinetic parameters. Both models were capable of depicting the full range of the fluoride breakthrough curves, revealing that the models are suitable tools to design fixed-bed systems using VPum and Zr–Pu. This study demonstrated that the coating with zirconium enhances the fluoride adsorption capacity of VPum and that the Zr–Pu could be a promising candidate for the removal of high levels of fluoride from groundwater at a technical scale. However, additional investigations on, for instance, the influence of competing ions, regeneration of fluoride-laden adsorbents, and technical and economic analysis are advisable to draw explicit conclusions.

## Figures and Tables

**Table 1:** Elemental and oxide compositions of natural pumice (VPum) and Zr–Pu.

Elemental Content	VPum % (wt)	Zr–Pu % (wt)	Oxide Content	VPum % (wt)	Zr–Pu % (wt)
Si	27.1	26.3	SiO_2_	68.9	63.7
Al	5.3	5.5	Al_2_O_3_	11.7	10.9
Fe	3.4	3.1	Fe_2_O_3_	6.7	5.4
K	3.8	3.4	K_2_O	5.5	4.3
Ca	0.3	0.4	CaO	1.1	0.2
Na	1.2	1.0	Na_2_O	1.9	2.1
Mg	0.1	0.1	MgO	0.1	-
Zn	<0.1	<0.1	TiO_2_	0.2	-
Zr	<0.1	3.9	ZrO_2_	-	8.9
Mn	<0.1	<0.1	MnO	0.1	0.2
Cr	<0.1	<0.1	ZnO	1.2	-
Cu	<0.1	<0.1	NiO	1.1	2.2
Co	<0.1	<0.1	CuO	1.6	1.7
Cd	<0.1	<0.1	-	-	-
Ni	<0.1	<0.1	-	-	-
Pb	<0.1	<0.1	-	-	-
As	<0.1	<0.1	-	-	-

**Table 2:** Textural properties of VPum and Zr–Pu.

Adsorbent	Specific Surface Area (m^2^/g)	Average Pore Size (nm)
VPum	3.45	4.43
Zr–Pu	9.63	3.52

**Table 3:** Fixed-bed column parameters obtained for defluoridation by zirconium–coated pumice (Zr–Pu) and on VPum (recent study) [5].

Parameter Studied	pH	C_O_ (mg/L)	Q_O_ (mL/min)	EBCT(min)	t_b_ (min)	t_e_ (min)	V_b_ (mL)	V_e_ (mL)	MTZ(cm)	q_b_ (mg/kg)	q_tot_ (mg)	q_e_ (mg/kg)	Adsorbent
Variation of pH keeping C_O_ and Q_O_ constant	2	10	1.25	412	3471	4781	4338.94	5976.76	2.74	163.18	59.78	224.78	Zr–Pu
	4	10	1.25	412	689	2058	861.25	2572.50	6.65	32.39	25.73	96.75	
	6	10	1.25	412	604	1469	755.00	1836.25	5.89	28.39	18.36	69.06	
	2	10	1.25	412	1206	2339	1507.50	2923.70	4.84	54.20	29.24	110.00	VPum
	4	10	1.25	412	278	500	347.50	625.00	4.44	13.00	6.25	23.51	
	6	10	1.25	412	135	315	168.75	393.75	5.71	6.40	3.94	14.81	
Variation of Q_O_ keeping pH and C_O_ constant	2	10	1.25	412	3471	4781	4338.94	5976.76	2.74	163.18	59.78	224.78	Zr–Pu
	2	10	2.50	206	1807	1360	2717.50	3400.00	2.00	102.20	34.00	127.87	
	2	10	3.75	137	557	768	2088.75	2880.00	2.75	78.55	28.80	108.31	
	2	10	1.25	412	1206	2339	1507.50	2923.70	4.84	54.20	29.24	110.00	VPum
	2	10	2.50	206	215	634	538.47	1585.16	6.60	20.30	7.93	29.80	
	2	10	3.75	137	75	359	282.69	1346.42	7.90	7.10	4.49	16.89	

t_b_ = breakthrough time, t_e_ = exhaustion time, V_b_ = total effluent volume at a breakthrough time, V_e_ = total effluent volume at exhaustion time MTZ = Mass Transfer Zone, EBCT = Empty Bed Contact Time, q_b_ = amount of fluoride removed at a breakthrough time per kg of adsorbent, q_total_ = total amount of fluoride adsorbed from the column, q_e_ = equilibrium fluoride uptake per kg of the adsorbent.

**Table 4:** Thomas model parameter values for defluoridation by Zr–Pu, and VPum [5].

Parameter Studied	pH	C_o_ (mg/L)	Q (mL/min)	Bed-Depth, H_B_ (cm)	K_T_ (L/min.mg) (×10^4^)	q_O(cal.)_ (mg/kg)	q_e(exp.)_ (mg/kg)	R^2^	RMSE	Adsorbent
Variation of pH keeping C_O_ and Q_O_ constant	2	10	1.25	10	1.110	226.31	224.78	0.994	0.0034	Zr–Pu
	4	10	1.25	10	1.270	93.54	96.75	0.986	0.0112	
	6	10	1.25	10	1.870	64.00	69.06	0.980	0.0082	
	2	10	1.25	10	1.440	110.00	110.00	0.993	0.0013	VPum
	4	10	1.25	10	8.289	17.83	23.51	0.977	0.0079	
	6	10	1.25	10	12.099	13.08	14.81	0.995	0.0034	
Variation of Q_O_ keeping pH and C_O_ constant	2	10	1.25	10	1.110	226.31	224.78	0.994	0.0034	Zr–Pu
	2	10	2.50	10	5.100	130.33	127.87	0.993	0.0047	
	2	10	3.75	10	10.000	78.07	108.31	0.992	0.0059	
	2	10	1.25	10	1.440	110.00	110.00	0.993	0.0013	VPum
	2	10	2.50	10	3.563	58.81	29.80	0.953	0.0167	
	2	10	3.75	10	5.000	45.82	16.90	0.962	0.0295	

**Table 5:** Adams-Bohart model parameter values for defluoridation by Zr–Pu, and VPum [5].

Parameter Studied	pH	C_o_ (mg/L)	Q (mL/min)	Bed-Depth, H_B_ (cm)	K_AB_ (L/min·mg) (×10^4^)	N_O(cal.)_ (mg/L)	R^2^	RMSE	Adsorbent
Variation of pH keeping C_O_ and Q_O_ constant	2	10	1.25	10	1.110	116.98	0.996	0.0034	Zr–Pu
	4	10	1.25	10	1.268	48.35	0.986	0.0112	
	6	10	1.25	10	1.874	33.08	0.980	0.0082	
	2	10	1.25	10	2.568	45.57	0.947	0.0013	VPum
	4	10	1.25	10	7.772	11.03	0.980	0.0079	
	6	10	1.25	10	11.220	7.75	0.980	0.0034	
Variation of Q_O_ keeping pH and C_O_ constant	2	10	1.25	10	1.110	116.98	0.996	0.0034	Zr–Pu
	2	10	2.50	10	5.063	69.31	0.993	0.0047	
	2	10	3.75	10	6.803	58.13	0.992	0.0059	
	2	10	1.25	10	2.568	45.57	0.947	0.0013	VPum
	2	10	2.50	10	4.813	30.03	0.957	0.0167	
	2	10	3.75	10	6.251	26.83	0.896	0.0295	

**Table 6:** Fluoride adsorption capacity of various adsorbents.

Adsorbents	Bed Height (cm)	Fluoride Level in (mg L^−1^)	Adsorption Capacity (mg g^−1^)	References
Granular acid-treated bentonite	28	6.34	0.190	[72]
Granular acid-treated bentonite	28	2.85	0.169	[72]
MnO_2_-coated Tamarind Fruit Shell	6	2	0.883	[71]
Aluminum modified iron oxide	10.5	4	0.139	[73]
Activated alumina (Grade OA-25)	10	5	0.74	[74]
Virgin Pumice (VPum)	10	10	0.110	[5]
Virgin Scoria (VSco)	10	10	0.022	[5]
Zr–Pu	10	10	0.225	This study

## Data Availability

The data used in this study are available from the authors at reasonable request.
